# Association between medication adherence and cardiovascular outcomes in patients with both diabetes and hypertension in primary care settings in Canada: A retrospective cohort study

**DOI:** 10.1371/journal.pone.0319991

**Published:** 2025-04-16

**Authors:** Min Su, Xiwen Simon Qin, Yanhong Li, Ross Upshur, Frank Sullivan, France Légaré, Michelle Greiver, Shishi Wu, Xiaolin Wei

**Affiliations:** 1 School of Public Administration, Inner Mongolia University, Hohhot, China; 2 Faculty of Pharmacology and Pharmacy, Hong Kong University, Hong Kong, China; 3 Laboratory of Data Discovery for Health (D24H), Hong Kong Science Technology Park, Hong Kong, China; 4 School of Population and Global Health, The University of Western Australia, Perth, Australia; 5 Dalla Lana School of Public Health, University of Toronto, Toronto, Canada; 6 Department of Family and Community Medicine, Faculty of Medicine, University of Toronto, Toronto, Canada; 7 School of Medicine, University of St Andrews, St Andrews, United Kingdom; 8 Faculty of Medicine, Laval University, Quebec, Quebec, Canada; 9 North York General Hospital, Toronto, Ontario, Canada; Tehran University of Medical Sciences, IRAN, ISLAMIC REPUBLIC OF

## Abstract

**Objectives:**

The impact of concurrent adherence to antihypertensives, antidiabetics, and statins on cardiovascular disease (CVD) outcomes and intermediate clinical outcomes in people with hypertension and diabetes remains unclear. This study aimed to evaluate the association between medication adherence and CVD outcomes in such patients.

**Methods:**

This retrospective cohort study analyzed the electronic medical records of 36,211 adults aged 18 or older diagnosed with hypertension and diabetes between January 2008 and June 2016 in Canada. Patients were prescribed antihypertensives, antidiabetics, and statins, with a minimum 1-year follow-up post-diagnosis. Medication adherence was determined by the proportion of days covered (PDC). For monotherapy, a PDC≥80% and <80% was reflected high and low adherence respectively. In multiple medication scenarios, adherence was considered high when each medication was at PDC≥80%; low when any medication fell below 80%. The primary outcome encompassed cardiac events, including coronary heart disease, stroke, and heart failure. Intermediate clinical outcomes included changes in diastolic blood pressure (DBP), Systolic Blood Pressure (SBP), glycated hemoglobin (HbA1c), low-density lipoprotein cholesterol (LDL-C), and total cholesterol (TC). Cox regression models assessed the association between medication adherence and CVD morbidity, all-cause mortality, and intermediate clinical outcomes.

**Results:**

High adherence to antidiabetic and statin monotherapy was associated with a lower all-cause mortality risk (aHR=0.67, P=0.001; aHR=0.68, P<0.001, respectively). For patients simultaneously prescribed three medications, higher adherence was linked to significant reductions in DBP (6 months: coefficient −0.52, P=0.01; 12 months: coefficient −0.44, P=0.02; 18 months: coefficient −0.55, P=0.004) and LDL-C (6 months: coefficient −0.04, P=0.02; 12 months: coefficient −0.05, P=0.01; 18 months: coefficient −0.04, P=0.02).

**Conclusions:**

High adherence to antidiabetic and statin monotherapy correlated with lower all-cause mortality risk and improved intermediate clinical outcomes. However, simultaneous adherence to three medications did not significantly affect CVD outcomes, but influenced intermediate outcomes. Therefore, improving adherence to antihypertensives, antidiabetics, and statins among patients with hypertension and diabetes is important in primary care settings.

## Introduction

The paradigm of chronic disease management is shifting toward the concurrent treatment of multiple comorbidities, such as diabetes and hypertension, as these conditions substantially contribute to multimorbidity in middle-aged and older populations. Previous epidemiological studies have reported that the occurrence of hypertension in diabetic patients is approximately twice as high as in those without diabetes [1,2]. Hypertension and diabetes are widely recognized as the primary factors contributing to an elevated risk of adverse outcomes in cardiovascular disease (CVD) and increased healthcare expenditure [3–6].

Clinical guidelines recommend adherence to pharmacotherapies that have been shown to reduce mortality and hospitalization rates in patients with hypertension and diabetes [7–13]. Specifically, in individuals with hypertension, adherence to anti-hypertensive medications is associated with a decreased risk of death and hospitalizations related to CVD, such as myocardial infarction, ischemic heart disease, stroke, unstable angina, coronary artery disease, and chronic heart failure [7–13]. Similarly, in people with diabetes, adherence to anti-diabetic medications, particularly metformin, is linked to a decrease in mortality and hospitalizations related to diabetes [14,15]. Moreover, statins have been proven effective in preventing CVD, particularly when used as part of therapy for patients with diabetes and hypertension [16–18]. However, nonadherence to statin medications among individuals with hypertension and diabetes is associated with increased CVD-related hospitalizations and poorer treatment outcomes [19–22].

Medication adherence is crucial for individuals with coexisting hypertension and diabetes who often require multiple medications to achieve optimal treatment outcomes. However, those subject to such polypharmacy may face challenges in adhering to their prescriptions, leading to reduced therapeutic efficacy, an increased risk of adverse health outcomes and increased healthcare costs [15,23–25]. Limited data exist on the impact of adherence to antihypertensive, antidiabetic, and statin medications in primary care. Understanding this impact is crucial for improving outcomes in patients with comorbid diabetes and hypertension, given the significant burden of these conditions in the primary care setting.

This study aimed to evaluate the impact of adherence to antihypertensive, antidiabetic, and statin medications on CVD events in individuals with both hypertension and diabetes. We hypothesized that 1) adherence to any or all of the specified medications is associated with a decreased risk of CVD events and all-cause mortality, and 2) adherence to any or all of the specified medications is associated with improvements in clinical indicators, including diastolic blood pressure (DBP), systolic blood pressure (SBP), glycated hemoglobin (HbA1c), low-density lipoprotein cholesterol (LDL-C), and total cholesterol (TC).

## Methods

This study was reported according to the research protocol [26] and the STROBE statement (see S1 Files for details).

### Ethics approval and consent to participate

The University of Toronto Health Science Center’s Ethics Committee granted ethics approval (approval number: 36065).

### Setting and data sources

This study leveraged the Diabetes Action Canadian National Diabetes Repository, which contains de-identified data from over 100,000 individuals with diabetes in four Canadian provinces (Ontario, Manitoba, Quebec, and Alberta). Data were retrieved from the electronic medical records (EMRs) of consenting family physicians and nurse practitioners via regional Practice-Based Research Networks. These networks were part of the Canadian Primary Care Sentinel Surveillance Network (CPCSSN), and data management followed established CPCSSN protocols. The data for this retrospective study were accessed on March 1, 2018. During and after data collection, the authors did not have access to information that could identify individual participants. For more detailed information, please refer to the protocol paper [26].

### Study design and participants

This retrospective cohort study included a cohort of 36,211 individuals aged ≥18 years who were diagnosed with both hypertension and diabetes between January 1, 2008, and June 31, 2016. To be included in the cohort, participants needed to have survived for at least 1 year after the diagnosis and have received prescriptions for antihypertensive, antidiabetic, and statin medications documented in their EMRs. Individuals who 1) had a history of CVD events (including acute coronary heart disease, stroke, and heart failure) prior to the diagnosis of diabetes or hypertension, 2) lacked information on the date of the CVD event, and 3) experienced a cardiovascular outcome before the landmark date (1 year after the diagnosis) were excluded.

### Exposure and study outcomes

#### Medication adherence.

The exposure variable was adherence to antihypertensive and antidiabetic agents and statins in patients with both diabetes and hypertension. Previous studies have demonstrated that the proportion of days covered (PDC) is a better measure of adherence than the medication possession ratio (MPR) or modified MPR (MPRm), as it is less prone to overestimating adherence levels and more reliable in predicting the relationship of adherence with outcome in survival analyses [27,28]. Therefore, in this study, the level of medication adherence was measured using the PDC, whichwas calculated using the following formula [27]:


$$\text{PDC}=\frac{Totaldayscoveredbydrugsupply}{Landmarkdate-1stsupplydate}$$ (1)

The PDC represents the proportion of days covered by the drug supply within the landmark period, from the initial medication dispensation to the landmark date, set at 1 year after the diagnosis of both diabetes and hypertension. For monotherapy, a PDC below 80% was considered to indicate low adherence, whereas a PDC of 80% or higher indicated high level. In scenarios involving multiple medications, high adherence was characterized by each medication achieving an 80% or greater PDC, whereas low adherence was defined as the PDC of any medication falling below 80%. Medication adherence was coded as Yes/No.

#### Outcomes of interest.

The primary outcomes assessed in this study were CVD events, defined by the World Health Organization (WHO) MONICA criteria. These events included acute coronary heart disease (ICD-9: 410–412, 414), stroke (ICD-9: 420–438), and heart failure (ICD-9: 428). All-cause mortality was another outcome in this study, representing the death rate calculated by considering all causes of death during the study period. Additionally, the incidence rate of the composite endpoint, including both CVD events and all-cause mortality, was calculated.

The study cohort was followed until the occurrence of the outcome, death, or end of the study (31 July 2016). The secondary outcomes were clinical treatment outcomes, including DBP, SBP, HbA1c, LDL-C, and TC.

### Covariates

Demographic characteristics were identified at the date of diagnosis, including age, sex, and body mass index (BMI). Risk factors, such as smoking history and alcohol consumption history, and baseline clinical outcomes, such as DBP, SBP, HbA1c, LDL-C, and TC, were also identified at baseline. Comorbidities, including chronic obstructive pulmonary disease (COPD), depression, dementia, and Parkinson’s disease, were identified at baseline. The data on all these variables came from the Diabetes Action Canadian National Diabetes Repository. Finally, all the covariates were time-fixed.

### Statistical analysis

The baseline characteristics were presented as counts and column percentages for categorical variables. For continuous variables that followed a normal distribution, the mean and standard deviation were provided. Subsequently, the incidence rate of the CVD events and all-cause mortality in the overall study population was calculated. To examine the impact of medication adherence on the primary and secondary outcomes, both univariable and multivariable Cox proportional-hazards models were employed [29,30]. Proportional hazards assumptions for the Cox model including all variables were assessed using Schoenfeld residuals. Hazard ratio (HR) estimates were considered statistically significant if the P-value was less than 0.05. The results were presented as adjusted HRs along with their corresponding 95% confidence intervals (CIs). Furthermore, the association between medication adherence and clinical outcomes (i.e., DBP, SBP, HbA1c, LDL-C, and TC) was examined using multivariable linear regression models. The statistical analyses were conducted using STATA (version 15, Stata Corp LP, College Station, Texas, USA).

## Results

### General characteristics of participants and medication adherence

We identified 36,211 patients with both diabetes and hypertension, with a mean age of 64.3 years (±12.5) and 47.3% being female. The mean BMI was 32.6 kg/m^2^ (±8.0). Most patients did not smoke (51.5%) or consume alcohol (66.5%). Approximately 5.3% of patients had the comorbidity of COPD, and 12.7% had the comorbidity of depression. Approximately 2.3% of patients had dementia, and 0.5% had Parkinson’s disease. Medication adherence to the three agents was 11.1%. More details can be found in Table 1.

**Table 1:** Baseline characteristics of study individuals.

Risk factors	Whole cohort (origin: diagnosis date of both diabetes and hypertension)		Whole cohort (origin: diagnosis date of both diabetes and hypertension + 1 year)			
			(Outcome: CVD)		(Outcome: Death)	
	Number of individuals (N=36,211)	Characteristics	Number of individuals (N=30,431)	Characteristics	Number of individuals (N=31,513)	Characteristics
Adherence						
No			26,763	88.9%	26,905	88.9%
Yes			3,344	11.1%	3,360	11.1%
Age (years)	35,900	64.4 (±12.8)	31,057	64.3 (±12.5)	31,232	64.3 (±12.5)
Sex						
Male	18,664	51.6%	16,499	52.7%	16,598	52.7%
Female	17,544	48.5%	14,835	47.3%	14,912	47.3%
Body Mass Index (BMI) (kg/m^2^)	26,017	32.5 (±8.0)	22,967	32.6 (±8.0)	23,090	32.6 (±8.0)
History of smoking						
No	13,133	51.4%	11,547	51.5%	11,587	51.5%
Yes	12,400	48.6%	10,869	48.5%	10,923	48.5%
History of alcohol						
No	16,814	65.9%	14,896	66.5%	14,949	66.4%
Yes	8,719	34.2%	7,520	33.6%	7,561	33.6%
Chronic obstructive pulmonary disease						
No	24,638	91.4%	29,677	94.7%	29,829	94.7%
Yes	2,319	8.6%	1,659	5.3%	1,684	53.4%
Depression						
No	22,976	82.1%	27,353	87.3%	27,510	87.3%
Yes	5,002	17.9%	3,983	12.7%	4,003	12.7%
Dementia						
No	25,293	95.0%	30,620	97.7%	30,788	97.7%
Yes	1,325	5.0%	716	2.3%	725	2.3%
Parkinson disease						
No	25,655	99.0%	31,183	99.5%	31,356	99.5%
Yes	249	1.0%	153	0.5%	157	0.5%
Diastolic blood pressure (DBP)	33,144	79.0 (±12.3)	28,950	78.8 (±12.2)	29,119	78.8 (±12.2)
Systolic blood pressure (SBP)	34,056	135.6 (±18.4)	29,656	135.4 (±18.3)	29,825	135.4 (±18.3)
Glycated haemoglobin (HbA1c)	28,251	6.7 (6.2, 7.6)	24,862	6.8 (6.2, 7.7)	24,954	6.8 (6.2, 7.7)
Low-density lipoprotein (LDL-C) cholesterol	28,362	2.2 (1.6, 3.0)	24,983	2.1 (1.6, 2.9)	25,069	2.1 (1.6, 2.9)
Total cholesterol (TC)	28,449	4.2 (3.5, 5.1)	25,091	4.1 (3.5, 5.0)	25,177	4.1 (3.5, 5.0)

Note: the numbers of participants with DBP, SBP, HbA1c, LDL-C, and TC were 33144, 34056, 28251, 28362, and 28,449 respectively, with the remaining data missing. As a result, the missing values were excluded from the analysis of the association between medication adherence and intermediate clinical outcomes.

### CVD outcomes and intermediate clinical outcomes in the overall study population

As shown in Table 2, the incidence rate of CVD was 3.55 per 1000 patients (95% CI=3.25–3.87). The incidence rate of all-cause mortality was 10.55 (95% CI=10.03–11.08). The incidence rate of the composite endpoint (including both CVD events and all-cause mortality) was 13.82 (95% CI=13.23–14.43).

**Table 2:** Incidence rate of CVD and all-cause of death.

Outcome	Number of individuals	Number of events	Median follow-up time (years)	Total person-years	Incidence rate (95%C.I.) (per 1000 individuals)
CVD	36，211	519	3.84 (2.05, 5.84)	146，336.14	3.55 (3.25, 3.87)
All-cause of mortality	36，211	1，557	3.88 (2.08,5.87)	147，649.83	10.55(10.03,11.08)
Composite endpoint (CVD events and all-cause mortality)	36，211	2，022	3.84 (2.05,5.84)	146，336.14	13.82(13.23,14.43)

The clinical outcomes at 6, 12, and 18 months of follow-up are shown in S1 Table. Notably, the mean SBP remained stable over the 18 months, with a slight decrease from 133.46 mmHg at 6 months to 133.25 mmHg at 18 months. Similarly, DBP showed a minimal decrease, from 77.72 mmHg at 6 months to 77.28 mmHg at 18 months. The mean HbA1c levels remained consistent throughout the follow-up period, averaging around 7.03% at both 6 and 12 months, with a slight increase to 7.04% at 18 months. The mean LDL-C and TC levels showed slight reductions from 6 to 18 months. More details can be found in S1 Table.

### Association between medication adherence and CVD events

Table 3 shows the univariate and multivariate aHRs (95% CI) for the effects of medication adherence on CVD events. High adherence to antihypertensive, antidiabetic, and statin monotherapy was not significantly associated with changes in the risk of CVD outcomes (aHR=0.89, 95% CI=0.70–1.15, P=0.37; aHR=0.95, 95% CI=0.76–1.18, P=0.64; aHR=0.78, 95%CI=0.58–1.05, P=0.09, respectively). Similarly, patients adherent to a combination of anti-diabetics, anti-hypertensives, and statins did not show significant differences in CVD risk to those with low medication adherence (aHR=0.71, 95% CI=0.46–1.11, P=0.13).

**Table 3:** Cox model for effect of risk factors on CVD risk.

Risk factors	Adherent (to antidiabetic mellitus medications)				Adherent (to antihypertension medications)				Adherent (to statins)				Adherent (to multiple drugs)			
	Univariable analysis		Multivariable analysis		Univariable analysis		Multivariable analysis		Univariable analysis		Multivariable analysis		Univariable analysis		Multivariable analysis	
	HR (95% C.I.)	P value	HR (95% C.I.)	P value	HR (95% C.I.)	P value	HR (95% C.I.)	P value	HR (95% C.I.)	P value	HR (95% C.I.)	P value	HR (95% C.I.)	P value	HR (95% C.I.)	P value
**Adherence (High vs. Low)**	**0.88 (0.68,1.13)**	**0.29**	**0.89 (0.70,1.15)**	**0.37**	**0.95 (0.76,1.18)**	**0.64**	**0.95 (0.76,1.18)**	**0.64**	**0.75 (0.55,1.00)**	**0.05**	**0.78 (0.58,1.05)**	**0.09**	**0.73 (0.47,1.12)**	**0.15**	**0.71 (0.46,1.11)**	**0.13**
Age (every 1 year increases)	1.05 (1.04,1.06)	<0.001	1.06 (1.05,1.07)	<0.001	1.06 (1.05,1.06)	< 0.001	1.06 (1.05,1.07)	< 0.001	1.05 (1.04,1.06)	< 0.001	1.06 (1.05,1.07)	< 0.001	1.05 (1.04,1.06)	< 0.001	1.06 (1.05,1.07)	< 0.001
Sex (Female vs. Male)	0.86 (0.71,1.04)	0.13	0.78 (0.64,0.95)	0.02	0.88 (0.72,1.08)	0.21	0.79 (0.63,0.97)	0.03	0.86 (0.71,1.05)	0.14	0.77 (0.63,0.95)	0.01	0.90 (0.70,1.14)	0.37	0.76 (0.59,0.97)	0.03
BMI (every 1 kg/m^2^ increases)	1.01 (1.00,1.03)	0.06	1.03 (1.02,1.05)	<0.001	1.01 (1.00,1.03)	0.06	1.04 (1.02,1.05)	< 0.001	1.02 (1.00,1.03)	0.02	1.04 (1.02,1.05)	< 0.001	1.02 (1.01,1.04)	0.001	1.04 (1.03,1.06)	< 0.001
History of smoking (Yes vs. No)	1.27 (0.98,1.63)	0.07	1.13 (0.84,1.51)	0.40	1.29 (0.98,1.69)	0.07	1.13 (0.82,1.54)	0.45	1.04 (0.80,1.36)	0.75	0.87 (0.60, 1.26)	0.44	1.18 (0.69,2.01)	0.51	1.01 (0.52,1.94)	0.98
History of alcohol (Yes vs. No)	1.19 (0.90,1.57)	0.21	1.09 (0.78,1.52)	0.59	1.25 (0.86,1.81)	0.21	1.14 (0.74,1.76)	0.51	1.21 (0.96,1.51)	0.10	1.27 (0.90,1.78)	0.16	1.23 (0.67,2.24)	0.45	1.21 (0.59,2.46)	0.55
COPD) (Yes vs. No)	1.99 (1.45,2.74)	<0.001	1.41 (1.02,1.94)	0.04	2.27 (1.65,3.12)	< 0.001	1.62 (1.17,2.24)	0.003	2.03 (1.47,2.79)	< 0.001	1.51 (1.09,2.08)	0.01	1.84 (1.19,2.84)	0.01	1.42 (0.92,2.21)	0.12
Depression (Yes vs. No)	1.06 (0.80,1.40)	0.70	1.13 (0.85,1.51)	0.39	1.04 (0.77,1.40)	0.80	1.11 (0.82,1.50)	0.50	0.94 (0.70,1.26)	0.69	1.03 (0.76,1.39)	0.85	1.12 (0.78,1.60)	0.55	1.24 (0.86,1.80)	0.25
Dementia (Yes vs. No)	1.23 (0.69,2.19)	0.47	0.60 (0.33,1.07)	0.09	1.16 (0.62,2.17)	0.65	0.52 (0.27,1.00)	0.05	1.03 (0.55,1.93)	0.92	0.52 (0.27,0.99)	0.05	0.58 (0.19,1.82)	0.35	0.33 (0.10,1.04)	0.06
Parkinson disease (Yes vs. No)	2.69 (1.20,6.03)	0.02	1.77 (0.78,4.01)	0.17	3.19 (1.42,7.15)	0.01	2.11 (0.93,4.80)	0.07	2.77 (1.24,6.21)	0.01	1.92 (0.85,4.34)	0.12	1.73 (0.43,6.96)	0.44	1.24 (0.31,5.04)	0.76
SBP (mmHg)																
>120 & SBP<=130 vs. <=120	0.82 (0.62,1.08)	0.16	0.86 (0.65,1.14)	0.29	0.92 (0.68,1.24)	0.58	0.97 (0.71,1.32)	0.85	0.85 (0.64,1.13)	0.26	0.90 (0.67,1.20)	0.48	1.10 (0.75,1.61)	0.63	1.15 (0.78,1.69)	0.49
>130 & SBP<=140 vs. <=120	0.82 (0.62,1.09)	0.17	0.88 (0.65,1.18)	0.38	0.89 (0.65,1.23)	0.48	0.96 (0.69,1.33)	0.79	0.84 (0.63,1.13)	0.25	0.90 (0.66,1.23)	0.52	1.20 (0.82,1.76)	0.36	1.24 (0.84,1.85)	0.28
>140 & SBP<=150 vs. <=120	0.81 (0.59,1.12)	0.20	0.88 (0.63,1.22)	0.44	0.84 (0.59,1.19)	0.32	0.89 (0.62,1.29)	0.55	0.85 (0.61,1.18)	0.33	0.91 (0.64,1.30)	0.61	1.17 (0.77,1.77)	0.47	1.22 (0.79,1.90)	0.37
>150 vs. <=120	0.94 (0.70,1.26)	0.67	1.03 (0.74,1.43)	0.86	1.13 (0.82,1.55)	0.47	1.24 (0.87,1.78)	0.24	1.02 (0.76,1.37)	0.90	1.14 (0.82,1.61)	0.44	1.68 (1.15,2.46)	0.01	1.83 (1.20,2.78)	0.01
DBP (every 1 mmHg increases)	0.998 (0.997,0.999)	< 0.001	1.000 (0.999,1.001)	0.48	0.998 (0.997,0.999)	< 0.001	1.000 (0.999,1.001)	0.36	0.998 (0.997,0.999)	< 0.001	0.999 (0.998,1.000)	0.14	0.999 (0.998,1.000)	0.01	0.999 (0.998,1.001)	0.30
HbA1c (every 1% increases)	1.02 (0.95,1.09)	0.62	1.06 (0.99,1.13)	0.09	0.99 (0.90,1.09)	0.85	1.03 (0.94,1.12)	0.50	0.99 (0.92,1.06)	0.76	1.02 (0.95,1.09)	0.54	1.00 (0.92,1.08)	0.94	1.04 (0.96,1.12)	0.34
LDL-C (every 1 mmol/L increases)	0.89 (0.77,1.03)	0.11	1.10 (0.94,1.28)	0.23	0.95 (0.86,1.05)	0.32	1.13 (0.97,1.32)	0.11	0.94 (0.76,1.15)	0.47	1.08 (0.92,1.28)	0.30	0.97 (0.87,1.09)	0.65	1.10 (0.94,1.28)	0.23
TC (every 1 mmol/L increases)	0.82 (0.76,0.89)	< 0.001	0.84 (0.75, 0.94)	< 0.001	0.86 (0.71,1.03)	0.09	0.86 (0.68,1.10)	0.19	0.88 (0.74,1.05)	0.13	0.90 (0.77,1.06)	0.18	0.93 (0.81,1.06)	0.26	0.93 (0.77,1.13)	0.45

### Association between medication adherence and all-cause mortality

As shown in Table 4, high adherence to monotherapy with antidiabetics and statins significantly reduced the risk of all-cause mortality (aHR=0.67, 95% CI=0.55–0.82, P=0.001; aHR=0.68, 95% CI=0.59–0.79, P<0.001, respectively). However, high adherence to antihypertensive medications was not significantly associated with a reduction in all-cause mortality. Similarly, patients with high adherence to a regimen combining antidiabetics, antihypertensives, and statins did not show significant changes in the risk of CVD outcomes (aHR=0.97, 95% CI=0.80–1.17, P=0.75).

**Table 4:** Cox model for effect of risk factors on all-cause mortality.

Risk factors	Adherent (to antidiabetic mellitus medications)				Adherent (to antihypertension medications)				Adherent (to statins)				Adherent (to multiple drugs)			
	Univariable analysis		Multivariable analysis		Univariable analysis		Multivariable analysis		Univariable analysis		Multivariable analysis		Univariable analysis		Multivariable analysis	
	HR (95% C.I.)	P value	HR (95% C.I.)	P value	HR (95% C.I.)	P value	HR (95% C.I.)	P value	HR (95% C.I.)	P value	HR (95% C.I.)	P value	HR (95% C.I.)	P value	HR (95% C.I.)	P value
**Adherence (Yes vs. No)**	**0.65 (0.53,0.81)**	**0.001**	**0.67 (0.55,0.82)**	**0.001**	**0.96 (0.84,1.08)**	**0.49**	**0.96 (0.84,1.09)**	**0.50**	**0.64 (0.55,0.74)**	**<0.001**	**0.68 (0.59,0.79)**	**< 0.001**	**0.99 (0.82,1.19)**	**0.93**	**0.97 (0.80,1.17)**	**0.75**
Age (every 1 year increases)	1.07 (1.06 1.07)	<0.001	1.06 (1.05,1.07)	<0.001	1.07 (1.07,1.08)	< 0.001	1.07 (1.06,1.07)	< 0.001	1.07 (1.07,1.08)	<0.001	1.07 (1.06,1.07)	< 0.001	1.08 (1.07,1.08)	< 0.001	1.07 (1.06,1.08)	<0.001
Sex (Female vs. Male)	0.82 (0.74,0.91)	<0.001	0.71 (0.63,0.79)	<0.001	0.83 (0.75,0.92)	< 0.001	0.71 (0.64,0.79)	< 0.001	0.82 (0.74,0.91)	<0.001	0.70 (0.63,0.78)	< 0.001	0.79 (0.71,0.89)	< 0.001	0.68 (0.61,0.77)	<0.001
BMI (every 1 kg/m^2^ increases)	0.98 (0.96,0.99)	0.01	1.01 (1.00,1.02)	0.17	0.97 (0.96,0.98)	< 0.001	1.00 (0.99,1.01)	0.40	0.98 (0.97,0.98)	<0.001	1.01 (1.00,1.02)	0.08	0.98 (0.97,0.99)	<0.001	1.01 (1.00,1.02)	0.20
History of smoking (Yes vs. No)	0.82 (0.69,0.98)	0.03	0.87 (0.72,1.04)	0.12	0.91 (0.82,1.01)	0.08	0.99 (0.87,1.13)	0.89	0.89 (0.72,1.09)	0.21	0.99 (0.80,1.23)	0.94	0.94 (0.83,1.07)	0.36	0.98 (0.84,1.15)	0.81
History of alcohol (Yes vs. No)	0.61 (0.51,0.72)	<0.001	0.61 (0.51,0.73)	<0.001	0.62 (0.54,0.72)	< 0.001	0.58 (0.49,0.69)	< 0.001	0.61 (0.52,0.73)	<0.001	0.57 (0.46,0.70)	< 0.001	0.68 (0.59,0.78)	<0.001	0.62 (0.51,0.75)	<0.01
COPD) (Yes vs. No)	2.90 (2.49,3.38)	<0.001	1.95 (1.67,2.28)	<0.001	2.79 (2.41,3.23)	< 0.001	1.85 (1.59,2.15)	< 0.001	2.79 (2.41,3.23)	<0.001	1.88 (1.61,2.18)	< 0.001	3.13 (2.64,3.71)	<0.001	2.10 (1.77,2.50)	<0.001
Depression (Yes vs. No)	1.11 (0.95,1.29)	0.18	1.21 (1.03,1.42)	0.02	1.09 (0.94,1.27)	0.23	1.20 (1.03,1.40)	0.02	1.10 (0.95,1.27)	0.21	1.22 (1.05,1.42)	0.01	1.19 (1.01,1.41)	0.04	1.33 (1.11,1.58)	0.001
Dementia (Yes vs. No)	4.34 (3.61,5.21)	<0.001	1.99 (1.63,2.42)	<0.001	4.20 (3.52,5.02)	< 0.001	1.80 (1.49,2.19)	< 0.001	4.23 (3.54,5.05)	<0.001	1.83 (1.51,2.22)	< 0.001	4.07 (3.28,5.06)	<0.001	1.78 (1.42,2.25)	<0.001
Parkinson disease (Yes vs. No)	2.60 (1.65,4.09)	<0.001	1.01 (0.64,1.61)	0.96	2.59 (1.67,4.03)	< 0.001	1.04 (0.66,1.63)	0.87	2.50 (1.61,3.88)	< 0.001	1.03 (0.65,1.61)	0.91	2.41 (1.39,4.16)	0.002	0.95 (0.54,1.67)	0.87
SBP (mmHg)																
>120 & SBP<=130 vs. <=120	0.76 (0.65,0.88)	<0.001	0.91 (0.78,1.06)	0.23	0.73 (0.63,0.84)	< 0.001	0.87 (0.75,1.01)	0.07	0.75 (0.64,0.86)	< 0.001	0.90 (0.77,1.04)	0.16	0.76 (0.64,0.89)	0.001	0.92 (0.78,1.10)	0.36
>130 & SBP<=140 vs. <=120	0.76 (0.65,0.89)	0.001	0.97 (0.82,1.15)	0.77	0.75 (0.64,0.87)	< 0.001	0.95 (0.81,1.11)	0.52	0.76 (0.66,0.88)	< 0.001	0.96 (0.82,1.12)	0.60	0.80 (0.67,0.94)	0.01	1.05 (0.88,1.24)	0.61
>140 & SBP<=150 vs. <=120	0.64 (0.53,0.77)	<0.001	0.87 (0.72,1.05)	0.14	0.66 (0.56,0.79)	< 0.001	0.88 (0.73,1.06)	0.18	0.67 (0.56,0.81)	< 0.001	0.87 (0.72,1.05)	0.14	0.64 (0.52,0.77)	< 0.001	0.86 (0.70,1.06)	0.17
>150 vs. <=120	0.82 (0.70,0.97)	0.02	1.23 (1.02,1.48)	0.03	0.83 (0.71,0.97)	0.02	1.19 (1.00,1.42)	0.05	0.83 (0.71,0.98)	0.03	1.19 (0.99,1.43)	0.07	0.84 (0.70,1.00)	0.06	1.27 (1.03,1.57)	0.03
DBP (every 1 mmHg increases)	0.996 (0.996,0.996)	<0.001	0.998 (0.998,0.999)	<0.001	0.996 (0.995,0.996)	< 0.001	0.998 (0.998,0.999)	< 0.001	0.996 (0.996,0.996)	< 0.001	0.998 (0.998,0.999)	< 0.001	0.996 (0.995,0.996)	< 0.001	0.998 (0.997,0.999)	< 0.001
HbA1c (every 1% increases)	1.00 (0.97,1.03)	0.97	1.06 (1.02,1.09)	0.001	0.99 (0.96,1.02)	0.35	1.04 (1.02,1.07)	0.003	0.98 (0.95,1.02)	0.34	1.04 (1.01,1.07)	0.01	0.99 (0.96,1.03)	0.62	1.05 (1.02,1.09)	0.001
LDL-C (every 1 mmol/L increases)	0.79 (0.73,0.84)	<0.001	0.93 (0.85,1.01)	0.10	0.79 (0.75,0.84)	< 0.001	0.96 (0.89,1.03)	0.26	0.80 (0.76,0.85)	< 0.001	0.97 (0.89,1.06)	0.50	0.76 (0.72,0.82)	< 0.001	0.95 (0.87,1.04)	0.28
TC (every 1 mmol/L increases)	0.83 (0.78,0.87)	<0.001	0.94 (0.88,1.01)	0.08	0.82 (0.78,0.86)	< 0.001	0.94 (0.89,1.00)	0.04	0.83 (0.79,0.87)	< 0.001	0.94 (0.88,1.00)	0.06	0.80 (0.76,0.84)	< 0.001	0.93 (0.86,1.00)	0.05

### Association between medication adherence and the composite endpoint

S2 Table shows the univariate and multivariate aHRs (95% CIs) for the effects of medication adherence on the composite endpoint. High adherence to antidiabetic and statin monotherapy was associated with a lower risk of the composite endpoint, including CVD events and all-cause mortality (aHR=0.81, 95% CI=0.70–0.94, P=0.01; aHR=0.79, 95%CI=0.67–0.92, P=0.003, respectively). High adherence to antihypertensive monotherapy was not significantly associated with the risk of the composite endpoint outcomes (aHR=0.91, 95% CI=0.82–1.02, P=0.10). Furthermore, patients with high adherence to combined antidiabetic, antihypertensive, and statin therapy did not experience significant changes in adverse outcomes (aHR=0.92, 95% CI=0.77–1.09, P=0.33).

### Association between medication adherence and intermediate clinical outcomes

Table 5 shows the effects of medication adherence on clinical outcomes. Adherence to antidiabetic monotherapy was significantly associated with reductions in DBP, LDL-C, and TC at 6, 12, and 18 months. Adherence to anti-hypertensive monotherapy was significantly associated with reductions in DBP and LDL-C at these time points. Adherence to statin monotherapy was significantly associated with reductions in DBP at 18 months and LDL-C at 6, 12, and 18 months. In patients concurrently prescribed the three medications, high adherence was significantly associated with reductions in DBP and LDL-C at 6, 12, and 18 months.

**Table 5:** Linear regression model for effect of risk factors on clinical outcomes.

Outcomes	Time points	Adherent (to antidiabetic mellitus medications)		Adherent (to antihypertension medications)		Adherent (to statins)		Adherent (to multiple drugs)	
		Coefficient (95%CI)	P value	Coefficient (95%CI)	P value	Coefficient (95%CI)	P value	Coefficient (95%CI)	P value
DBP	at 6 months	-0.33 (-0.49, -0.18)	<0.001	-0.10 (-0.22, 0.01)	0.08	-0.05 (-0.19, 0.09)	0.49	-0.52 (-0.90, -0.14)	0.01
	at 12 months	-0.35 (-0.49, -0.20)	<0.001	0.08 (-0.04, 0.19)	0.18	-0.02 (-0.16, 0.12)	0.79	-0.44 (-0.83, -0.06)	0.02
	at 18 months	-0.43 (-0.58, -0.29)	<0.001	-0.22 (-0.33, -0.11)	<0.001	-0.33 (-0.48, -0.19)	<0.001	-0.55 (-0.94, -0.17)	0.004
SBP	at 6 months	0.44 (0.22, 0.67)	<0.001	1.14 (0.96, 1.32)	<0.001	0.41 (0.18, 0.64)	0.001	0.17 (-0.43, 0.76)	0.58
	at 12 months	0.27 (0.04, 0.49)	0.02	1.37 (1.18, 1.55)	<0.001	0.60 (0.37, 0.83)	<0.001	0.13 (-0.47, 0.73)	0.67
	at 18 months	0.39 (0.16, 0.62)	0.001	0.84 (0.65, 1.02)	<0.001	0.02 (-0.21, 0.25)	0.87	-0.02 (-0.63, 0.59)	0.95
HbA1c	at 6 months	0.19 (0.17, 0.22)	<0.001	0.09 (0.06, 0.11)	<0.001	0.12 (0.10, 0.15)	<0.001	-0.02 (-0.09, 0.05)	0.54
	at 12 months	0.18 (0.15, 0.20)	<0.001	0.07 (0.05, 0.09)	<0.001	0.10 (0.08, 0.13)	<0.001	-0.01 (-0.08, 0.06)	0.79
	at 18 months	0.20 (0.16, 0.23)	<0.001	0.06 (0.04, 0.09)	<0.001	0.09 (0.07, 0.12)	<0.001	0.00 (-0.07, 0.07)	0.91
LDL-C	at 6 months	-0.03 (-0.04, -0.01)	<0.001	-0.04 (-0.05, 0.03)	<0.001	-0.04 (-0.06, -0.03)	<0.001	-0.04 (-0.08, 0.01)	0.02
	at 12 months	-0.04 (-0.05, -0.02)	<0.001	-0.30 (-0.04, -0.02)	<0.001	-0.04 (-0.06, -0.03)	<0.001	-0.05 (-0.08, 0.01)	0.01
	at 18 months	-0.03 (-0.05, -0.02)	<0.001	-0.04 (-0.05, 0.03)	<0.001	-0.04 (-0.05, -0.02)	<0.001	-0.04 (-0.08, 0.01)	0.02
TC	at 6 months	-0.02 (-0.04, 0.00)	0.01	-0.01 (-0.02, 0.00)	0.17	0.01 (-0.01, 0.03)	0.55	0.01 (-0.04, 0.06)	0.64
	at 12 months	-0.03 (-0.04, -0.01)	0.001	0.00 (-0.01, 0.01)	0.94	0.01 (-0.01, 0.03)	0.48	0.01 (-0.04, 0.06)	0.66
	at 18 months	-0.03 (-0.04, -0.01)	0.004	0.00 (-0.02, 0.01)	0.63	0.03 (0.00, 0.05)	0.02	0.02 (-0.03, 0.07)	0.49

## Discussion

To our knowledge, this is the first study to explore the association between adherence to antihypertensives, antidiabetics, and statins and CVD outcomes in primary care settings. Our findings indicate that strengthening adherence to monotherapies involving antidiabetic medications and statins is associated with all-cause mortality risk reductions of 33% and 32%, respectively. However, concurrent adherence to all three medications did not significantly influence CVD risk, although it did affect intermediate clinical outcomes. This study underscores the importance of streamlining medication regimens for patients with both diabetes and hypertension, to improve adherence among those prescribed multiple drugs concurrently. This strategy may facilitate the improvement of long-term health outcomes.

We found that the adherence rate of the three studied medications (11.1%) was lower than that reported previously. For example, numerous reviews show that adherence among patients suffering from chronic diseases averages about 50% in developed countries [31]. A 5-year cohort study from Hong Kong involving 218,047 newly diagnosed hypertensive individuals, that applied the PDC metric, found that 32.9% had poor adherence (PDC<40%), 12.1% had intermediate adherence (40–79%), and 55.0% exhibited high adherence (≥80%) [32]. In South Korea, a retrospective cohort study of 65,067 patients diagnosed with type 2 diabetes mellitus showed an adherence rate (≥80%) of 34.2% using the PDC method [33]. Furthermore, in the United States, a study of 155,597 newly diagnosed hypertensive patients, aged 66 to 79 years, revealed poor adherence (PDC<40%) in 8.9%, intermediate adherence (40–79%) in 30.3%, and high adherence (≥80%) in 60.8% of patients [10]. One plausible explanation for these variations is that the previous studies primarily focused on adherence to a single medication, whereas our study examined adherence to three different medications. Previous research has consistently established a robust link between medication regimen complexity and adherence among patients with diabetes and hypertension [33–36]. Crowley et al. (2014) found a significant association between higher medication regimen complexity scores and lower adherence rates in type 2 diabetes patients [35]. Likewise, Krousel-Wood et al. (2010) demonstrated an inverse relationship between medication burden and adherence among hypertensive patients [36]. These findings highlight the need to consider the influence of regimen complexity on medication adherence.

Our study also revealed that patients adhering to antidiabetic and statin monotherapy had a reduced risk of all-cause mortality and improved intermediate clinical outcomes, which aligns with previous studies [33,34]. Our findings, along with earlier research, also highlighted an increased risk of coronary events, ischemic stroke, unstable angina pectoris, and myocardial infarction among diabetes mellitus or hypertension patients with adherence below 80%, compared to those with a PDC of at least 80% [35–37]. For instance, in a retrospective cohort study of 65,067 individuals newly diagnosed with type 2 diabetes mellitus (aged ≥40 years) in South Korea, diabetic patients with the lowest PDC (<0.2) had an elevated risk for CVD (aHR=1.41, 95% CI=1.30–1.52, P< 0.001) compared to the highest PDC group (≥0.8) [33]. Similarly, for CVD mortality with a PDC<0.20, the aHR ranged from 1.36 to 1.54 compared to that for a PDC≥0.80 [33]. Similarly, a study in the United States, which included 155,597 newly diagnosed hypertensive patients aged 66 to 79 years, observed that a PDC greater than 80% significantly reduced the risk of CVD outcomes (aHR=0.44, 95% CI=0.42–0.45), including ischemic heart disease, stroke/transient ischemic attack, and heart failure [10]. However, these studies did not explore medication adherence among patients diagnosed with both diabetes and hypertension concurrently. Our study fills this gap by showing that simultaneous adherence to three medications in patients with both conditions did not significantly reduce CVD risk but could impact intermediate clinical outcomes. The possible reason for the non-significant impact on CVD outcomes is that the complexity of the medication may influence patient adherence, thereby affecting their health outcomes. Therefore, comprehending the factors contributing to this complexity and implementing strategies to streamline regimens are imperative for enhancing adherence rates and overall patient care [38]. By incorporating approaches like combination therapy, fixed-dose combinations, medication synchronization, patient education, and medication reviews to reduce the complexity of medication regimens, healthcare providers can assist patients in effectively managing their conditions and improving medication adherence and treatment outcomes [39,40].

This study has the following strengths. First, it addressed an evidence gap by exploring medication adherence among patients diagnosed with both diabetes and hypertension and examining the synergistic benefits of adhering to multiple medications in preventing CVD events and mortality. Second, we used PDC as the measurement to define the exposure variable, medication adherence, and treated as it time-varying in the analysis. The PDC is a widely measured parameter in pharmacy claim databases and is prevalently used across studies to prevent overestimation [28]. However, it is often treated as a fixed-time variable, which may cause the nuances of changing prescription patterns over time to be overlooked [41,42]. This approach may introduce bias into the link between medication use and clinical outcomes due to variations in dosing behaviors between patients with erratic and consistent medication intake, potentially resulting in divergent clinical outcomes. To overcome this limitation, we employed a time-varying PDC method [42], which accommodates dynamic prescription patterns during the follow-up period. This approach helps to mitigate information bias arising from changes in medication regimens, such as switches or discontinuations, thereby providing a more accurate estimation of the association between medication adherence and outcomes.

Our study also has the following limitations. First, the study design carries the risk of errors and omissions. For example, CVD cases could have been incompletely captured and underestimated. Given that primary care physicians are responsible for diagnosing diabetes, hypertension, and CVD, delays in EMR inputs might have occurred. Second, while our analysis accounted for individual-level risk factors and environmental determinants, the database does not include a range of variables that could influence patients’ understanding of their treatment and daily management, such as primary language, ethnicity, health literacy, employment status, and marital status. Additionally, essential process variables, such as a patient’s involvement in treatment decision-making, disease comprehension, family and social support, and physician-patient communication regarding prescription benefits and adverse effects, are not captured in the database. Furthermore, variables from the healthcare system angle, including access to primary care and primary care models, are absent. Importantly, community health support, including treatment assistance and regular visits to general practitioners, bears significance but is not encompassed. Finally, a limitation of this study is the presence of missing data for key clinical measures, including DBP, SBP, HbA1c, LDL-C, and TC. Participants with missing data were excluded from the analysis of the association between medication adherence and intermediate clinical outcomes, which may affect the generalizability of the results. These limitations may have introduced bias into our findings.

## Conclusion

Our study showed that high adherence to monotherapy with antidiabetics and statins was associated with a lower risk of all-cause mortality, and intermediate clinical outcomes. However, simultaneous adherence to the three medications did not significantly change CVD outcomes but impacted intermediate outcomes. Evidently, the complexity of medication regimens can influence patients’ adherence behaviors, consequently affecting treatment outcomes. This study underscores the significance of streamlining medication regimens for patients grappling with both diabetes and hypertension, offering a notable avenue for enhancing long-term health outcomes. Adherence to antihypertensive medications, antidiabetics, and statins needs to be improved in primary care settings. Our findings may help identify challenges to medication adherence faced by diabetic and hypertensive patients in primary care settings and support the development of intervention strategies that promote medication adherence among patients with multi-morbidities.

## Supporting information

S1 File STROBE statement. (DOC)

S1 Table Mean and median for lab outcome. (DOCX)

S2 Table Cox model for effect of risk factors on composite endpoint (including CVD risk and all-cause mortality). (DOCX)

## References

[1] Haile TG, Mariye T, Tadesse DB, Gebremeskel GG, Asefa GG, Getachew T. Prevalence of hypertension among type 2 diabetes mellitus patients in Ethiopia: a systematic review and meta-analysis. Int Health. 2023;15(3):235–41. doi: 10.1093/inthealth/ihac060 36055967 PMC10153558

[2] Nouh F, Omar M, Younis M. Prevalence of Hypertension among Diabetic Patients in Benghazi: A Study of Associated Factors. AJMAH. 2017;6(4):1–11. doi: 10.9734/ajmah/2017/35830

[3] Liu Q, Quan H, Chen G, Qian H, Khan N. Antihypertensive medication adherence and mortality according to ethnicity: a cohort study. Can J Cardiol. 2014;30(8):925–31. doi: 10.1016/j.cjca.2014.04.017 25064583

[4] Osterberg L, Blaschke T. Adherence to medication. N Engl J Med. 2005;353(5):487–97. doi: 10.1056/NEJMra050100 16079372

[5] Chobanian AV, Bakris GL, Black HR, Cushman WC, Green LA, Izzo JL Jr, et al. The Seventh Report of the Joint National Committee on Prevention, Detection, Evaluation, and Treatment of High Blood Pressure: the JNC 7 report. JAMA. 2003;289(19):2560–72. doi: 10.1001/jama.289.19.2560 12748199

[6] Poulter NR, Borghi C, Parati G, Pathak A, Schmieder RE. Medication adherence in hypertension. Journal of Hypertension. 2019;38(4). 10.1097/HJH.0000000000002294 31834123

[7] Karlsson SA, Hero C, Svensson A-M, Franzén S, Miftaraj M, Gudbjörnsdottir S, et al. Association between refill adherence to lipid-lowering medications and the risk of cardiovascular disease and mortality in Swedish patients with type 2 diabetes mellitus: a nationwide cohort study. BMJ Open. 2018;8(3):e020309. doi: 10.1136/bmjopen-2017-020309 29602853 PMC5884334

[8] Kim J-A, Kim E-S, Lee E-K. Evaluation of the chronic disease management program for appropriateness of medication adherence and persistence in hypertension and type-2 diabetes patients in Korea. Medicine (Baltimore). 2017;96(14):e6577. doi: 10.1097/MD.0000000000006577 28383439 PMC5411223

[9] Shin S, Song H, Oh S-K, Choi KE, Kim H, Jang S. Effect of antihypertensive medication adherence on hospitalization for cardiovascular disease and mortality in hypertensive patients. Hypertens Res. 2013;36(11):1000–5. doi: 10.1038/hr.2013.85 23966057

[10] Yang Q, Chang A, Ritchey MD, Loustalot F. Antihypertensive Medication Adherence and Risk of Cardiovascular Disease Among Older Adults: A Population-Based Cohort Study. J Am Heart Assoc. 2017;6(6):e006056. doi: 10.1161/JAHA.117.006056 28647688 PMC5669200

[11] Perreault S, Dragomir A, White M, Lalonde L, Blais L, Bérard A. Better adherence to antihypertensive agents and risk reduction of chronic heart failure. J Intern Med. 2009;266(2):207–18. doi: 10.1111/j.1365-2796.2009.02084.x 19623691

[12] Roy L, White-Guay B, Dorais M, Dragomir A, Lessard M, Perreault S. Adherence to antihypertensive agents improves risk reduction of end-stage renal disease. Kidney Int. 2013;84(3):570–7. doi: 10.1038/ki.2013.103 23698228

[13] Wu P-H, Yang C-Y, Yao Z-L, Lin W-Z, Wu L-W, Chang C-C. Relationship of blood pressure control and hospitalization risk to medication adherence among patients with hypertension in Taiwan. Am J Hypertens. 2010;23(2):155–60. doi: 10.1038/ajh.2009.210 19927135

[14] Simard P, Presse N, Roy L, Dorais M, White-Guay B, Räkel A, et al. Association Between Metformin Adherence and All-Cause Mortality Among New Users of Metformin: A Nested Case-Control Study. Ann Pharmacother. 2018;52(4):305–13. doi: 10.1177/1060028017743517 29144162

[15] Asche C, LaFleur J, Conner C. A review of diabetes treatment adherence and the association with clinical and economic outcomes. Clin Ther. 2011;33(1):74–109. doi: 10.1016/j.clinthera.2011.01.019 21397776

[16] Grundy SM, Stone NJ, Bailey AL, Beam C, Birtcher KK, Blumenthal RS, et al. 2018 AHA/ACC/AACVPR/AAPA/ABC/ACPM/ADA/AGS/APhA/ASPC/NLA/PCNA Guideline on the Management of Blood Cholesterol: Executive Summary: A Report of the American College of Cardiology/American Heart Association Task Force on Clinical Practice Guidelines. J Am Coll Cardiol. 2019;73(24):3168–209. Epub 20181110. doi: 10.1016/j.jacc.2018.11.002. PubMed . 30423391 10.1016/j.jacc.2018.11.002

[17] Cho Y, Jeong Y, Seo DH, Ahn SH, Hong S, Suh YJ, et al. Use of statin for the primary prevention of cardiovascular outcomes in elderly patients: A propensity-matched cohort study. Atherosclerosis. 2021;328:92–9. doi: 10.1016/j.atherosclerosis.2021.05.022 34126505

[18] Lin Y-W, Wang C-C, Wu C-C, Hsu Y-T, Lin F-J. Effectiveness of statins for the primary prevention of cardiovascular disease in the Asian elderly population. Int J Cardiol. 2023;373:25–32. doi: 10.1016/j.ijcard.2022.11.031 36435332

[19] Aubert RE, Yao J, Xia F, Garavaglia SB. Is there a relationship between early statin compliance and a reduction in healthcare utilization? Am J Manag Care. 2010;16(6):459–66. 20560689

[20] Pittman DG, Chen W, Bowlin SJ, Foody JM. Adherence to statins, subsequent healthcare costs, and cardiovascular hospitalizations. Am J Cardiol. 2011;107(11):1662–6. doi: 10.1016/j.amjcard.2011.01.052 21439533

[21] Rannanheimo PK, Tiittanen P, Hartikainen J, Helin-Salmivaara A, Huupponen R, Vahtera J, et al. Impact of Statin Adherence on Cardiovascular Morbidity and All-Cause Mortality in the Primary Prevention of Cardiovascular Disease: A Population-Based Cohort Study in Finland. Value Health. 2015;18(6):896–905. doi: 10.1016/j.jval.2015.06.002 26409618

[22] Bonaccio M, Di Castelnuovo A, Costanzo S, Persichillo M, De Curtis A, Cerletti C, et al. Interaction between Mediterranean diet and statins on mortality risk in patients with cardiovascular disease: Findings from the Moli-sani Study. Int J Cardiol. 2019;276:248–54. doi: 10.1016/j.ijcard.2018.11.117 30527993

[23] K J, Rao M, Yn S, Thunga G, N R, Sudhakar C, et al. Determinants of Medication Non-Adherence Among the Elderly with Co-Existing Hypertension and Type 2 Diabetes Mellitus in Rural Areas of Udupi District in Karnataka, India. Patient Prefer Adherence. 2023;17:1641–56. doi: 10.2147/PPA.S380784 37465058 PMC10351531

[24] Bingham JM, Black M, Anderson EJ, Li Y, Toselli N, Fox S, et al. Impact of Telehealth Interventions on Medication Adherence for Patients With Type 2 Diabetes, Hypertension, and/or Dyslipidemia: A Systematic Review. Ann Pharmacother. 2021;55(5):637–49. doi: 10.1177/1060028020950726 32815400

[25] Baiee HA, Makai MB. Medication Adherence in Hypertensive Diabetic Patients. Medical Journal of Babylon. 2022;19(4):569–74. doi: 10.4103/mjbl.mjbl_129_22

[26] Su M, Haldane V, Upshur R, Sullivan F, Légaré F, Greiver M, et al. The Impact of Treatment Adherence for Patients With Diabetes and Hypertension on Cardiovascular Disease Risk: Protocol for a Retrospective Cohort Study, 2008-2018. JMIR Res Protoc. 2019;8(5):e13571. doi: 10.2196/13571 31152529 PMC6658229

[27] Qin X, Hung J, Knuiman MW, Briffa TG, Teng T-HK, Sanfilippo FM. Comparison of medication adherence measures derived from linked administrative data and associations with mortality using restricted cubic splines in heart failure patients. Pharmacoepidemiol Drug Saf. 2020;29(2):208–18. doi: 10.1002/pds.4939 31958191

[28] Bijlsma MJ, Janssen F, Hak E. Estimating time-varying drug adherence using electronic records: extending the proportion of days covered (PDC) method. Pharmacoepidemiol Drug Saf. 2016;25(3):325–32. doi: 10.1002/pds.3935 26687394

[29] Wei X, Zou G, Gong W, Yin J, Yu Y, Walley J, et al. Cardiovascular disease risk reduction in rural China: a clustered randomized controlled trial in Zhejiang. Trials. 2013;14:354. doi: 10.1186/1745-6215-14-354 24160442 PMC4015636

[30] Talic S, Marquina C, Ofori-Asenso R, Petrova M, Liew D, Owen AJ, et al. Switching, Persistence and Adherence to Statin Therapy: a Retrospective Cohort Study Using the Australian National Pharmacy Data. Cardiovasc Drugs Ther. 2022;36(5):867–77. doi: 10.1007/s10557-021-07199-7 34097194

[31] Burkhart PV, Sabaté E. Adherence to long-term therapies: evidence for action. J Nurs Scholarsh. 2003;35(3):207. doi: 10.1111/j.1547-5069.2003.tb00001.x 14562485

[32] Wong MCS, Tam WWS, Cheung CSK, Wang HHX, Tong ELH, Sek ACH, et al. Drug adherence and the incidence of coronary heart disease- and stroke-specific mortality among 218,047 patients newly prescribed an antihypertensive medication: a five-year cohort study. Int J Cardiol. 2013;168(2):928–33. doi: 10.1016/j.ijcard.2012.10.048 23174167 PMC7114239

[33] Kim Y-Y, Lee J-S, Kang H-J, Park SM. Effect of medication adherence on long-term all-cause-mortality and hospitalization for cardiovascular disease in 65,067 newly diagnosed type 2 diabetes patients. Sci Rep. 2018;8(1):12190. doi: 10.1038/s41598-018-30740-y 30111867 PMC6093904

[34] Cross AJ, Elliott RA, Petrie K, Kuruvilla L, George J. Interventions for improving medication-taking ability and adherence in older adults prescribed multiple medications. Cochrane Database Syst Rev. 2020;5(5):CD012419. doi: 10.1002/14651858.CD012419.pub2 32383493 PMC7207012

[35] Crowley MJ, Holleman R, Klamerus ML, Bosworth HB, Edelman D, Heisler M. Factors associated with persistent poorly controlled diabetes mellitus: clues to improving management in patients with resistant poor control. Chronic Illn. 2014;10(4):291–302. doi: 10.1177/1742395314523653 24567193 PMC4317345

[36] Krousel-Wood MA, Islam T, Muntner P, Stanley E, Phillips A, Webber LS, et al. Medication adherence in older clinic patients with hypertension after Hurricane Katrina: implications for clinical practice and disaster management. Am J Med Sci. 2008;336(2):99–104. doi: 10.1097/MAJ.0b013e318180f14f 18703901 PMC2561300

[37] Ho PM, Magid DJ, Masoudi FA, McClure DL, Rumsfeld JS. Adherence to cardioprotective medications and mortality among patients with diabetes and ischemic heart disease. BMC Cardiovasc Disord. 2006;6:48. doi: 10.1186/1471-2261-6-48 17173679 PMC1762024

[38] Scott IA, Hilmer SN, Reeve E, Potter K, Le Couteur D, Rigby D, et al. Reducing inappropriate polypharmacy: the process of deprescribing. JAMA Intern Med. 2015;175(5):827–34. doi: 10.1001/jamainternmed.2015.0324 25798731

[39] Gokoel SRM, Gombert-Handoko KB, Zwart TC, van der Boog PJM, Moes DJAR, de Fijter JW. Medication non-adherence after kidney transplantation: A critical appraisal and systematic review. Transplant Rev (Orlando). 2020;34(1):100511. doi: 10.1016/j.trre.2019.100511 31627978

[40] Maxwell LG, McFarland MS, Baker JW, Cassidy RF. Evaluation of the Impact of a Pharmacist-Led Telehealth Clinic on Diabetes-Related Goals of Therapy in a Veteran Population. Pharmacotherapy. 2016;36(3):348–56. doi: 10.1002/phar.1719 26877253

[41] Dalli LL, Kilkenny MF, Arnet I, Sanfilippo FM, Cummings DM, Kapral MK, et al. Towards better reporting of the proportion of days covered method in cardiovascular medication adherence: A scoping review and new tool TEN-SPIDERS. Br J Clin Pharmacol. 2022;88(10):4427–42. doi: 10.1111/bcp.15391 35524398 PMC9546055

[42] Hunter KB, Glickman ME, Campos LF. Inferring medication adherence from time-varying health measures. Stat Med. 2022;41(12):2205–26. doi: 10.1002/sim.9351 35137428

