# Supplementary material for: Association between medication adherence and cardiovascular outcomes in patients with both diabetes and hypertension in primary care settings in Canada: A retrospective cohort study
Source: PLoS One. 2025 Apr 16;20(4):e0319991. doi: 10.1371/journal.pone.0319991 (PMC12002471; doi:10.1371/journal.pone.0319991)
Supplement: S1 Table — (DOCX) [file pone.0319991.s002.docx]

Table S1 Mean and median for lab outcome

| Outcome |  | Mean (± SD) | Median (IQR^#^ ) |
| --- | --- | --- | --- |
| Diastolic blood pressure (DBP) | at 6 months | 77.72 (±11.67) | 78 (70, 84) |
|  | at 12 months | 77.47 (±11.59) | 78 (70, 84) |
|  | at 18 months | 77.28 (±11.64) | 78 (70, 84) |
| Systolic blood pressure (SBP) | at 6 months | 133.46 (±17.31) | 131 (122, 142) |
|  | at 12 months | 133.43 (±17.40) | 131 (122, 142) |
|  | at 18 months | 133.25 (±17.27) | 131 (122, 142) |
| Glycated haemoglobin (HbA1c) | at 6 months | 7.03 (±1.77) | 6.7 (6.1, 7.5) |
|  | at 12 months | 7.03 (±1.78) | 6.7 (6.1, 7.5) |
|  | at 18 months | 7.04 (±1.77) | 6.7 (6.1, 7.5) |
| Low-density lipoprotein cholesterol (LDL-C) | at 6 months | 2.32 (±1.13) | 2.10 (1.58, 2.87) |
|  | at 12 months | 2.29 (±1.14) | 2.07 (1.56, 2.82) |
|  | at 18 months | 2.27 (±1.13) | 2.04 (1.53, 2.80) |
| Total cholesterol (TC) | at 6 months | 4.36 (±1.53) | 4.12 (3.45, 5.00) |
|  | at 12 months | 4.33 (±1.52) | 4.08 (3.43, 4.97) |
|  | at 18 months | 4.31 (±1.53) | 4.05 (3.40, 4.94) |

Note: # IQR means interquartile range (25th percentile, 75th percentile).
