# Supplementary material for: Association between medication adherence and cardiovascular outcomes in patients with both diabetes and hypertension in primary care settings in Canada: A retrospective cohort study
Source: PLoS One. 2025 Apr 16;20(4):e0319991. doi: 10.1371/journal.pone.0319991 (PMC12002471; doi:10.1371/journal.pone.0319991)
Supplement: S2 Table — (DOCX) [file pone.0319991.s003.docx]

Table S2 Cox model for effect of risk factors on composite endpoint (including CVD risk and all-cause mortality)

| Risk factors | Adherent (to antidiabetic mellitus medications) | | | | Adherent (to antihypertension medications) | | | | Adherent (to Statins) | | | | Adherent (to multiple drugs) | | | |
| --- | --- | --- | --- | --- | --- | --- | --- | --- | --- | --- | --- | --- | --- | --- | --- | --- |
|  | Univariable analysis | | Multivariable analysis | | Univariable analysis | | Multivariable analysis | | Univariable analysis | | Multivariable analysis | | Univariable analysis | | Multivariable analysis | |
|  | HR (95% C.I.) | P value | HR (95% C.I.) | P value | HR (95% C.I.) | P value | HR (95% C.I.) | P value | HR (95% C.I.) | P value | HR (95% C.I.) | P value | HR (95% C.I.) | P value | HR (95% C.I.) | P value |
| Adherence  (Yes vs. No) | 0.77  (0.66,0.90) | 0.003 | 0.81  (0.70,0.94) | 0.01 | 0.91 (0.82,1.00) | 0.06 | 0.91 (0.82,1.02) | 0.10 | 0.75  (0.64,0.87) | 0.001 | 0.79  (0.67,0.92) | 0.003 | 0.94  (0.79,1.11) | 0.46 | 0.92  (0.77,1.09) | 0.33 |
| Age  (every 1 year increases) | 1.07 (1.06,1.07) | <0.001 | 1.06  (1.06,1.07) | <0.001 | 1.07 (1.06,1.07) | <0.001 | 1.06  (1.06,1.07) | <0.001 | 1.07 (1.06,1.07) | <0.001 | 1.06  (1.06,1.07) | < 0.001 | 1.07  (1.07,1.08) | < 0.001 | 1.07 (1.06,1.07) | < 0.001 |
| Sex  (Female vs. Male) | 0.82  (0.75,0.89) | <0.001 | 0.71 (0.65,0.78) | <0.001 | 0.83  (0.76,0.91) | <0.001 | 0.72  (0.66,0.79) | <0.001 | 0.82  (0.75,0.90) | <0.001 | 0.71  (0.65,0.78) | < 0.001 | 0.80  (0.72,0.89) | < 0.001 | 0.69  (0.62,0.77) | < 0.001 |
| BMI  (every 1 kg/m^2^ increases) | 0.99 (0.98,0.99) | <0.001 | 1.01 (1.01,1.02) | <0.001 | 0.99  (0.98,0.99) | 0.004 | 1.02 (1.01,1.02) | 0.001 | 0.99  (0.98,1.00) | 0.02 | 1.02  (1.00,1.03) | 0.01 | 0.99  (0.98,1.00) | 0.01 | 1.02 (1.01,1.02) | 0.001 |
| History of smoking  (Yes vs. No) | 0.92  (0.79,1.07) | 0.25 | 0.95  (0.76,1.19) | 0.62 | 0.93 (0.80,1.07) | 0.26 | 0.96 (0.82,1.12) | 0.57 | 0.93 (0.83,1.04) | 0.21 | 0.97  (0.86,1.09) | 0.64 | 0.99  (0.87,1.13) | 0.88 | 0.99  (0.85,1.14) | 0.85 |
| History of alcohol  (Yes vs. No) | 0.73  (0.59,0.91) | 0.01 | 0.71 (0.52,0.97) | 0.04 | 0.73 (0.64,0.84) | <0.001 | 0.71  (0.61,0.83) | <0.001 | 0.75 (0.64,0.87) | 0.001 | 0.71  (0.60,0.84) | 0.001 | 0.78  (0.64,0.94) | 0.01 | 0.73 (0.59,0.90) | 0.01 |
| COPD)  (Yes vs. No) | 2.70  (2.36,3.08) | <0.001 | 1.85 (1.62,2.12) | <0.001 | 2.60  (2.27,2.97) | <0.001 | 1.78  (1.55,2.03) | <0.001 | 2.60 (2.27,2.97) | <0.001 | 1.79  (1.57,2.06) | < 0.001 | 2.88  (2.45,3.39) | < 0.001 | 2.01 (1.70,2.37) | < 0.001 |
| Depression  (Yes vs. No) | 1.06 (0.93, 1.20) | 0.41 | 1.14 (0.99, 1.30) | 0.07 | 1.03 (0.90, 1.18) | 0.65 | 1.13 (0.98, 1.29) | 0.09 | 1.03  (0.91,1.18) | 0.62 | 1.13  (0.99,1.30) | 0.07 | 1.16  (0.99,1.36) | 0.06 | 1.28 (1.09,1.50) | 0.003 |
| Dementia  (Yes vs. No) | 3.47 (2.93, 4.11) | < 0.001 | 1.60 (1.33, 1.92) | < 0.001 | 3.45 (2.92, 4.09) | < 0.001 | 1.56 (1.31, 1.87) | < 0.001 | 3.47 (2.93,4.10) | < 0.001 | 1.58  (1.32,1.89) | < 0.001 | 3.48  (2.81,4.30) | < 0.001 | 1.61 (1.29,2.02) | < 0.001 |
| Parkinson disease  (Yes vs. No) | 2.53  (1.71,3.76) | <0.001 | 1.12  (0.74,1.69) | 0.59 | 2.54 (1.71,3.76) | < 0.001 | 1.17 (0.77,1.77) | 0.46 | 2.44  (1.65,3.63) | < 0.001 | 1.14  (0.76,1.71) | 0.51 | 2.05  (1.19,3.54) | 0.01 | 0.91 (0.52,1.59) | 0.75 |
| SBP (mmHg) |  |  |  |  |  |  |  |  |  |  |  |  |  |  |  |  |
| >120 & SBP<=130  vs. <=120 | 0.79  (0.69, 0.89) | <0.001 | 0.92  (0.80,1.04) | 0.19 | 0.77  (0.68,0.88) | < 0.001 | 0.88 (0.77,1.01) | 0.07 | 0.78 (0.68,0.89) | < 0.001 | 0.91  (0.80,1.04) | 0.16 | 0.79  (0.68,0.92) | 0.002 | 0.92  (0.79,1.08) | 0.33 |
| >130 & SBP<=140  vs. <=120 | 0.79  (0.70,0.90) | 0.001 | 0.98  (0.85,1.12) | 0.73 | 0.78 (0.68,0.89) | < 0.001 | 0.95 (0.83,1.08) | 0.44 | 0.79  (0.70,0.91) | 0.001 | 0.97  (0.84,1.11) | 0.64 | 0.86 (0.74,1.01) | 0.06 | 1.06  (0.91,1.25) | 0.46 |
| >140 & SBP<=150  vs. <=120 | 0.70  (0.60,0.82) | <0.001 | 0.89  (0.76,1.05) | 0.16 | 0.70  (0.60,0.81) | < 0.001 | 0.86 (0.74,1.01) | 0.07 | 0.71  (0.61,0.83) | < 0.001 | 0.88  (0.75,1.03) | 0.11 | 0.70 (0.58,0.83) | < 0.001 | 0.88 (0.73,1.07) | 0.21 |
| >150 vs. <=120 | 0.88  (0.77,1.01) | 0.07 | 1.20  (1.03,1.40) | 0.02 | 0.89 (0.77,1.02) | 0.09 | 1.20 (1.03,1.39) | 0.02 | 0.89  (0.78,1.03) | 0.12 | 1.21  (1.04,1.41) | 0.02 | 0.95 (0.80,1.12) | 0.53 | 1.33  (1.10,1.61) | 0.003 |
| DBP  (every 1 mmHg increases) | 0.996 (0.996,0.997) | < 0.001 | 0.998  (0.998,0.999) | < 0.001 | 0.996  (0.996, 0.997) | < 0.001 | 0.998  (0.998, 0.999) | < 0.001 | 0.996  (0.996,0.997) | < 0.001 | 0.998  (0.998,0.999) | < 0.001 | 0.996 (0.996,0.997) | < 0.001 | 0.998  (0.998,0.999) | < 0.001 |
| HbA1c  (every 1% increases) | 1.00  (0.97,1.03) | 0.83 | 1.05  (1.02,1.08) | <0.001 | 0.99 (0.96,1.02) | 0.54 | 1.04  (1.01,1.07) | 0.003 | 1.00 (0.97,1.03) | 0.89 | 1.05  (1.02,1.08) | 0.002 | 1.00  (0.96,1.03) | 0.88 | 1.06  (1.02,1.09) | 0.002 |
| LDL-C  (every 1 mmol/L increases) | 0.82  (0.78,0.86) | <0.001 | 0.99  (0.93,1.05) | 0.64 | 0.81  (0.76,0.86) | < 0.001 | 0.97  (0.90,1.05) | 0.41 | 0.83  (0.78,0.87) | < 0.001 | 0.98  (0.91,1.05) | 0.52 | 0.80  (0.75,0.87) | < 0.001 | 0.98  (0.90,1.06) | 0.60 |
| TC  (every 1 mmol/L increases) | 0.83  (0.80,0.87) | <0.001 | 0.93  (0.88,0.98) | 0.01 | 0.83  (0.79,0.87) | < 0.001 | 0.94 (0.89,0.99) | 0.03 | 0.85 (0.81,0.88) | < 0.001 | 0.95  (0.90,1.01) | 0.08 | 0.83 (0.78,0.88) | < 0.001 | 0.93  (0.87,0.99) | 0.03 |
